# Association Between Brain Care Score and Cognitive Performance: Findings From a Community‐Based Cohort in Singapore

**DOI:** 10.1002/brb3.71449

**Published:** 2026-05-08

**Authors:** Li Feng Tan, Jasper R. Senff, Shuna Shiann Khoo, Sanjula Singh, Reshma A. Merchant, Yee Wei Lim, Leonard Lee, Christopher D. Anderson, Jonathan Rosand, Lile Jia, Benjamin Y. Q. Tan, the Health District @ Queenstown

**Affiliations:** ^1^ Healthy Ageing Program Alexandra Hospital Singapore Singapore; ^2^ McCance Center for Brain Health Massachusetts General Hospital Boston Massachusetts USA; ^3^ Department of Neurology Mass General Brigham Boston Massachusetts USA; ^4^ Department of Psychology Faculty of Arts and Social Sciences National University of Singapore Singapore Singapore; ^5^ Department of Medicine National University of Singapore Singapore Singapore; ^6^ Yong Loo Lin School of Medicine National University of Singapore Singapore Singapore; ^7^ Lloyd's Register Foundation Institute for the Public Understanding of Risk (IPUR) and NUS Business School National University of Singapore Singapore Singapore; ^8^ Division of Neurology Department of Medicine National University Hospital Singapore Singapore

## Abstract

**Objectives:**

: The Brain Care Score (BCS) is a recently developed tool that measures modifiable risk factors for brain health. This study evaluated the association between BCS and cognitive performance in a community‐based cohort from Singapore.

**Methods:**

: Data were drawn from the Health District @ Queenstown baseline study, a representative multiethnic community cohort. The BCS (range 0–21; higher scores = healthier profiles) was derived from baseline questionnaires across physical, lifestyle, and socioemotional domains. Cognitive recall was assessed with the five‐item word recall test, and executive function with the Eriksen flanker task.

**Results:**

: A total of 5224 participants (mean age 52.8 ± 17.5 years; 53.1% women) were surveyed between September 2023 and May 2024. Participants with impairment had lower mean BCS than those without (14.9 ± 3.1 vs. 16.2 ± 2.8; *p* < 0.001). Each one‐point BCS increase was linked to 5% lower odds of impaired recall (odds ratios [OR] 0.95; 95% confidence intervals [CI] 0.92–0.98; *p* = 0.002), whereas per five‐point higher BCS corresponded to 23% lower odds (OR 0.77; 95% CI 0.66–0.91; *p* = 0.002). Each one‐point BCS increase was also associated with a 0.03‐point higher executive function score (*β* 0.03; 95% CI 0.01–0.05; *p* = 0.02), with per five‐point higher BCS yielding a 0.15‐point higher score (*β* 0.15; 95% CI 0.03–0.27; *p* = 0.02). Associations were stronger among younger, Chinese, and higher income participants.

**Conclusion:**

: Higher BCS was associated with better cognitive performance. These findings support its use as a potential community‐based tool for brain health risk assessment in Asian populations.

## Graphical abstract

In a community‐based Singapore cohort (n = 5, 224), higher Brain Care Score (BCS), reflecting modifiable brain health risk factors, is associated with better cognitive performance. Each incremental increase in BCS corresponds to lower odds of impaired memory and improved executive function. Findings support BCS as a practical tool for population‐level brain health assessment and risk stratification.

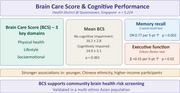

## 1 Introduction

Dementia is a leading contributor to disease burden worldwide, as highlighted by the 2021 Global Burden of Disease Study, which shows a rising trend in disability‐adjusted life‐years (DALYs) (GBD 2021 Nervous System Disorders Collaborators 2024). The 2024 update of the Lancet Commission on dementia highlights strong and growing evidence that addressing many of these risk factors throughout life—particularly in midlife—can reduce the risk of neurocognitive disease, increase healthy years of life, and emphasizes that it is never too early or too late to adopt risk‐reduction lifestyle changes (Livingston et al. 2024).

However, there remains a notable lack of comprehensive screening tools to effectively assess risk factors to prevent or delay dementia and enhance brain health (Woo 2015). This gap also limits our ability to proactively enhance brain health—a concept that goes beyond the absence of disease. The American Academy of Neurology's (AAN) Brain Health Initiative defines brain health as a continuous state of attaining and maintaining the optimal neurologic function that best supports an individual's physical, mental, and social well‐being through every stage of life (Rost et al. 2023). The WHO recommends taking advantage of protective strategies to assure brain health (World Health Organization 2022). As the global population ages, shifting the focus from late‐stage disease management to lifelong brain health promotion is increasingly recognized as a public health priority. By embedding dementia risk reduction within a broader brain health framework, there is an opportunity to take a proactive, life‐course approach—identifying risks early, supporting healthy behaviors, and enabling individuals to sustain cognitive vitality well into older age.

The Brain Care Score (BCS) is a recently developed, evidence‐based, and pragmatic tool created in partnership with patients (Singh et al. 2023). The BCS was developed through a modified Delphi process (Hasson et al. 2000) with feedback from both patients and practitioners. Designed as a simple, actionable measure, the BCS aims to motivate risk factor modification, shifting the paradigm from reactive treatment to proactive brain health promotion (Singh et al. 2022). The BCS ranges from 0 to 21 and consists of four physical components (blood pressure, hemoglobin A1c, cholesterol, and body mass index [BMI]), five lifestyle elements (nutrition, alcohol intake, smoking, aerobic activities, and sleep), and three social factors (stress, relationships, and purpose in life). A five‐point higher BCS was associated with a 14% lower risk of incident dementia (hazard ratio [HR]: 0.86, 95% confidence intervals [CI]: 0.81–0.91, *p* value: <0.001) (Singh et al. 2023). However, no studies have yet examined its application in Asian populations, despite the increasing prevalence of neurocognitive diseases in this region, driven by rapidly aging populations (Wu et al. 2015).

In this cross‐sectional study, we investigated the associations between the BCS and cognitive performance in a population‐based cohort of community‐dwelling adults in Singapore. We hypothesize that a higher BCS is associated with better cognitive performance.

## 2 Methods

### 2.1 Study Cohort and Recruitment

The baseline study for the Health District @ Queenstown sampled a community‐based multiethnic cohort comprising residents from the Queenstown district in Singapore. To ensure a representative sample, the Department of Statistics (DOS) Singapore provided a selection of postal codes and addresses, reflecting the district's demographics (age, race, and housing type). Invitation letters were sent to these addresses, and banners and posters were displayed in public areas. Trained interviewers then visited the home addresses to offer participation in a questionnaire and a series of physical health measurements. Only Singaporean citizens and permanent residents aged 21 and above were eligible, with up to two individuals per household invited. Data collection occurred between September 2023 and May 2024 (*N* = 5224), with written informed consent obtained from all participants. The study was approved by the Institutional Review Board of the National University of Singapore (NUS‐IRB‐2023‐297).

Data collected included sociodemographic characteristics, physical, lifestyle, and social‐emotional risk factors. The five‐item recall test based on the Montreal Cognitive Assessment (MoCA) was used to assess impaired cognitive recall (Nasreddine et al. 2005). Participants who recalled two words or less were categorized as having an impaired cognitive performance (Li et al. 2018). Executive function was assessed using the Ericksen flanker test (EFT). EFT measures conflict processing and has been used to evaluate executive cognitive function in patients with dementia and neurodegenerative diseases (Wang et al. 2013; Luks et al. 2010). EFT responses were cleaned using a binning procedure that combined speed and accuracy into a single score (Draheim et al. 2016). Responses below 200 ms were first removed, followed by those that were more than three standard deviations (SD) from an individual's mean reaction time (Draheim et al. 2016). An interference effect that captures how quickly a participant responds accurately on each incongruent trial compared to his/her response on accurate congruent trials was calculated. The interference effects across all participants were rank‐ordered and categorized into 10 deciles. A bin value from 1 (fastest 10%) to 10 (slowest 10%) was assigned to each decile. Additionally, a bin value of 20 was assigned for each inaccurate incongruent trial. Lastly, the mean bin score across all incongruent trials was computed and recoded such that a higher bin score reflects better performance. Poorer performance in EFT has been found in persons with mild cognitive impairment (MCI) and Alzheimer's dementia (AD), and EFT has been touted as a possible quick screening tool for AD (Ho et al. 2019).

### 2.2 Computation of BCS

The BCS was adapted for the Queenstown cohort on the basis of the questionnaire responses. For BCS components without a direct equivalent in the dataset, the most appropriate proxy measures were selected through author consensus (BYQT and JRS). Asian‐specific BMI cutoff values were applied (World Health Organization 2000), and local nutrition guidelines informed dietary scoring (WHO Expert Consultation 2004; Ng et al. 2023; Health Promotion Board 2025). Several clinical biomarkers were not directly measured in this community cohort; therefore, selected BCS domains—namely, blood pressure, cholesterol, diabetes, nutrition quality, sleep, stress, and meaning in life—were assessed using validated self‐report proxies. Other domains (BMI, smoking, alcohol intake, physical activity, and social relationships) were assessed directly or using closely aligned questionnaire measures. All original BCS domains were retained, and scoring thresholds were harmonized to maintain comparability with the original framework. The total adapted BCS therefore continued to range from 0 to 21. A detailed comparison between the original BCS and the Queenstown‐adapted BCS is provided in Table S1. There were no missing data for the exposure (BCS), cognitive outcomes (recall and executive function), or covariates included in the regression models. Accordingly, all 5224 participants were retained in the primary analyses.

### 2.3 Statistical Analysis

Participant characteristics are presented as counts and proportions, means and SD or medians and interquartile ranges (IQR), as appropriate. Continuous variables were compared using *t*‐tests for normally distributed data and Mann–Whitney *U* tests for non‐normal data. Normality was evaluated using histograms.

We examined the association between the BCS and cognitive performance using regression models. For recall, a dichotomized outcome variable was analyzed using logistic regression. For executive function, measured as a continuous variable, we applied linear regression. Our main exposure compares per five‐point difference in baseline BCS among individuals, aligning with previous studies (Singh et al. 2023, 2024; Rivier et al. 2024). A five‐point increase in BCS is considered to be an achievable yet meaningful improvement in someone's brain care. A five‐point increase can be achieved by, for example: (i) lowering blood pressure and reducing alcohol consumption or (ii) quitting smoking and reducing stress. All regression models were adjusted for a priori selected confounders, including age, sex, ethnicity, education, and income, similar to previously described methodology. Effect sizes are presented as odds ratios (OR) or beta (*β*) coefficients with 95% CI. To obtain an incremental estimation of the BCS, we performed 10,000 simulations of the primary regression models to generate estimates of the effect size of the BCS with the median and mean as a reference (King et al. 2000). In each simulation, the BCS regression coefficient was randomly drawn from a normal distribution defined by its estimated beta and standard error from the fitted logistic model. OR were then calculated across the BCS range (0–21) relative to the reference value, and the 2.5th and 97.5th percentiles of the simulated distribution were used to derive 95% CI. The reference values correspond to the overall sample mean and median of total BCS in the analytic cohort, and the same reference values were applied in all stratified analyses to ensure comparability across groups. The selection of the mean and median was intended to provide clinically interpretable reference points within the observed distribution. The simulations assume a linear association between total BCS and the log‐odds of cognitive decline, consistent with the specification of the logistic regression model, and no alternative functional forms were imposed in subgroup analyses.

### 2.4 Sensitivity Analysis

In sensitivity analysis, we stratified the regression models for a priori selected baseline characteristics that could influence the association between the BCS and the outcomes. We stratified based on (i) age (dichotomized at 60 years), (ii) ethnicity (categorized as [a] Chinese, [b] Malay, and [c] Indian), (iii) education level (categorized as [a] below high school, [b] high school graduate, and [c] college education and above, and (iv) household income (categorized as [a] 0–3999 Singaporean Dollar [SGD], [b] 4000–10,000 [SGD], [c] ≥10,000 [SGD], and [d] uncertain).

A two‐tailed *p* value of <0.05 was considered statistically significant. All analyses were performed using RStudio Version 3.3.0. The study was conducted in accordance with the Strengthening the Reporting of Observational Studies in Epidemiology (STROBE) guidelines.

## 3 Results

The baseline demographics of the study population are presented in Table 1. A total of 5224 residents in Queenstown were surveyed. The proportion of women (53.1%) and men (46.9%) and distribution of ethnicities were reflective of the national population. The mean age of participants was 52.8 ± 17.5 years. Among comorbidities, hypertension was the most prevalent, affecting 22.8% of participants (*N* = 1189), followed by hyperlipidemia in 21.8% (*N* = 1144). Diabetes was present in 10.6% (*N* = 559), and ischemic heart disease was reported by 5.2% (*N* = 275) (Table 1). The median BCS score was 16 (IQR: 14–18), and the overall BCS distribution is shown in Figure S1.

**TABLE 1:** Baseline characteristics of participants.

	Overall (*n* = 5224)
Age, years	52.8 (17.5)
Gender	
Male	2451 (46.9)
Female	2773 (53.1)
Ethnicity	
Chinese	3728 (71.4)
Malay	653 (12.5)
Indian	717 (13.7)
Others	126 (2.4)
BMI, kg/m^2^	24.2 ± 5.6
Education	
Below secondary	851 (16.3)
Secondary	986 (18.9)
Postsecondary	3387 (64.8)
Monthly household income	
0–3999 SGD	1504 (28.8)
4000–10,000 SGD	1611 (30.8)
≥10,000 SGD	2109 (40.4)
Comorbidities	
Hypertension	1189 (22.8)
Hyperlipidemia	1144 (21.8)
Diabetes	559 (10.6)
Heart disease	275 (5.2)
Stroke	52 (1.0)
Cancer	116 (2.2)

Abbreviation: BMI, body mass index.

### 3.1 Impaired Cognitive Recall

**FIGURE 1:**
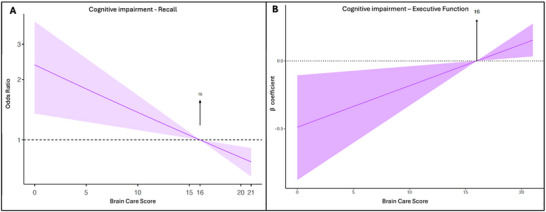
**Association between brain care score and impaired cognitive recall and executive function**. Regression curves of the BCS with the mean as reference and shade as 95% CI. The *Y*‐axis presents odds ratio (A) or *β* coefficient (B), and the *X*‐axis presents the Brain Care Score. (A) Impaired cognitive recall and (B) impaired executive function.

On the basis of the five‐item recall test, 517 (9.9%) were categorized as having impaired cognitive recall. There was a significant difference in mean BCS between participants with impaired cognitive recall and participants without (mean BCS 14.9 ± 3.1 vs. 16.2 ± 2.8, *p* < 0.001). Multivariate logistic regression was performed to assess the association between BCS and odds of impaired cognitive recall. Each one‐point increase in BCS was associated with a 5% reduction in the odds of impairment (OR 0.95, 95% CI 0.92–0.98; *p* = 0.002), whereas per five‐point higher BCS was associated with a 23% reduction in odds (OR 0.77, 95% CI 0.66–0.91; *p* = 0.002) (Table 2). The dose–response relationship between BCS and impaired cognitive recall is illustrated in Figure 1, Panel A.

**TABLE 2:** Logistic regression of impaired cognitive recall and impaired executive function with BCS scores.

**Outcome**	**Regression model^a^**	**Variable**	**OR (95% CI)**	***p* value**
Impaired cognitive recall	Logistic regression	BCS 1 point	0.95 (0.92–0.98)	0.002
		BCS 5 points	0.77 (0.66–0.81)	0.002
Impaired executive function	Linear regression	BCS 1 point	0.03 (0.01–0.05)	0.02
		BCS 5 points	0.15 (0.03–0.27)	0.02

Abbreviations: BCS, Brain Care Score; CI, confidence interval; OR, odds ratio.

^a^Adjusted for sex, age, ethnicity, education, and income.

### 3.2 Executive Function

In the executive function domain, each one‐point increase in BCS was associated with a 0.03‐point improvement in EFT performance (*β* 0.03, 95% CI 0.01–0.05; *p* = 0.02). Similarly, a five‐point difference in BCS was associated with a 0.15‐point improvement in EFT score (*β* 0.15, 95% CI 0.03–0.27; *p* = 0.02) (Table 2). The dose–response relationship between BCS and executive function is illustrated in Figure 1, Panel B.

### 3.3 Sensitivity Analyses for Impaired Cognitive Recall

**FIGURE 2:**
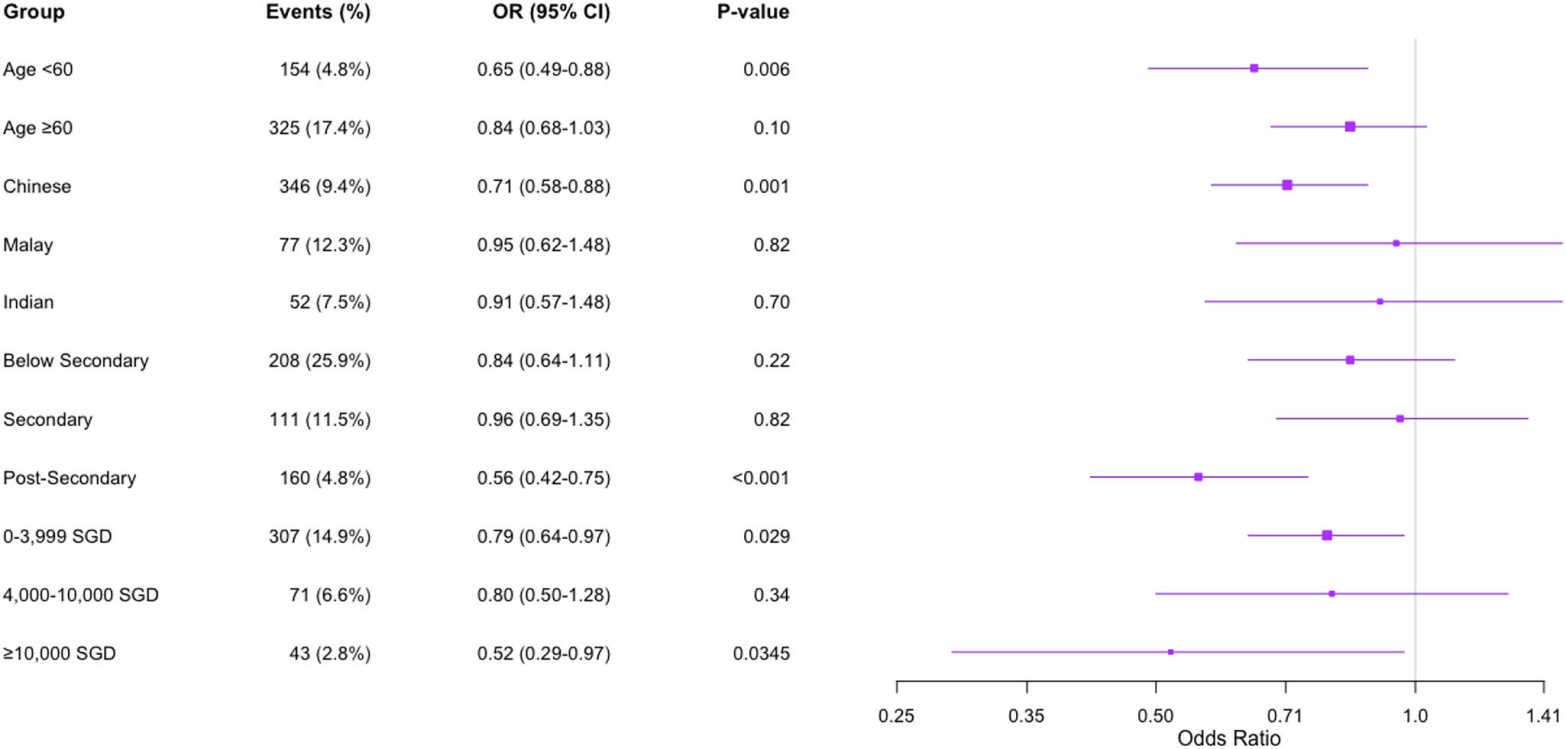
Stratified sensitivity analyses of impaired cognitive recall by demographic subgroups per five‐point increase in Brain Care Score. CI, confidence intervals.

Sensitivity analyses were performed to assess potential factors that could influence the observed relationship between BCS and impaired cognitive recall (Figure 2) and executive function. These analyses were stratified by key demographic variables (age, ethnicity, education, and income) to assess whether associations appeared broadly consistent across subgroups; formal interaction testing was not undertaken. Accordingly, subgroup findings should be interpreted cautiously and considered exploratory rather than confirmatory. Age appeared to modify the association, with younger participants (<60 years) exhibiting lower odds of impairment per five‐point higher BCS (OR 0.65, 95% CI 0.49–0.88; *p* < 0.001) compared to those aged 60 and above (OR 0.84, 95% CI 0.68–1.03; *p* = 0.10). Ethnic differences were also evident, as Chinese participants demonstrated reduced odds of impairment per five‐point higher BCS (OR 0.71, 95% CI 0.58–0.88; *p* = 0.001). In contrast, no significant difference was detected for Malay participants (OR 0.95, 95% CI 0.65–1.48; *p* = 0.82) or Indian participants (OR 0.91, 95% CI 0.57–1.48; *p* = 0.70).

Educational attainment and income levels also appeared to influence the relationship. Individuals with postsecondary education exhibited the lowest odds of impaired cognitive recall per five‐point higher BCS (OR 0.56, 95% CI 0.42–0.75; *p* < 0.001), whereas those with secondary (OR 0.96, 95% CI 0.69–1.35; *p* = 0.82) or below‐secondary education (OR 0.84, 95% CI 0.64–1.11; *p* = 0.22) showed no significant differences. Individuals in the lowest income bracket (SGD 0–3999) had significantly lower odds of impairment per five‐point higher BCS (OR 0.79, 95% CI 0.64–0.97; *p* = 0.029), as well as those in the highest (SGD ≥ 10,000) income bracket (OR 0.52, 95% CI 0.29–0.97; *p* = 0.035). However, findings were less consistent across middle‐income groups, suggesting variability in the potential modifying role of socioeconomic status.

### 3.4 Sensitivity Analyses for Executive Function

**FIGURE 3:**
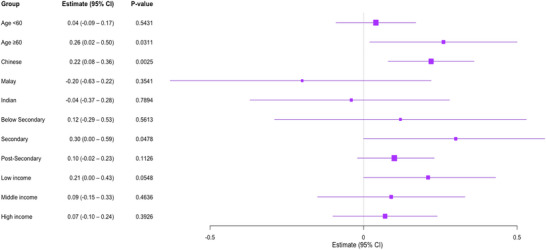
Stratified sensitivity analyses of impaired executive function by demographic subgroups per five‐point increase in Brain Care Score. CI, confidence intervals.

For executive function, demographic patterns showed some similarities to cognitive recall findings, though fewer associations achieved statistical significance. Significant associations between BCS and EFT performance were observed among participants aged 60 or older (*β* 0.26, 95% CI 0.02–0.50; *p* = 0.031) and those of Chinese ethnicity (*β* 0.22, 95% CI 0.08–0.36, *p* = 0.003) (Figure 3). These findings suggest that higher BCS was associated with modestly better executive function performance in these subgroups. Differences in baseline BCS distributions and risk factor profiles across subgroups may partly explain these patterns.

### 3.5 BCS Domains and Disparities Across Quartiles

Figure S2 shows the distribution of BCS domains across quartiles. Among individual BCS components, the greatest disparities between the top and bottom quartiles were observed in blood pressure, BMI, diet, and smoking (Figure S3).

## 4 Discussion

This study demonstrates the significant association between BCS and cognitive performance in a community‐based cohort from Singapore. The BCS was adapted for our population, and higher BCS scores were associated with lower odds of impaired cognitive performance. This protective effect was observed in both younger and older age subgroups (<60 and >60 years), with a more pronounced effect in the younger cohort.

Our findings corroborate international evidence and recommendations that addressing major risk factors for cognitive impairment early (the earlier, the better), at mid‐life (Zhang et al. 2022) and across one's life course may help to significantly reduce dementia risk (Livingston et al. 2024). Specifically in our cohort, the BCS identified that physical and lifestyle factors are key areas to focus population health strategies on. Studies have shown that a healthy dietary pattern is associated with a lower risk of cognitive impairment later in life (Tong et al. 2021; Hosking et al. 2014; Wright et al. 2017). A large population‐based study (Tong et al. 2021) found that those who demonstrated an improvement in diet quality had a significantly lower likelihood of cognitive impairment in an incremental manner. Even those in the lowest 40% of diet quality at baseline, moderate improvements in diet quality were associated with lower risk of cognitive impairment. This highlights the importance of encouraging improvements in diet and other risk factors over one's life course even for those who start at a low baseline. Other studies (Zhou et al. 2014; Hagger‐Johnson et al. 2013) have found that the coexistence of smoking and regular alcohol intake at midlife had a much stronger impact than the individual factors on risk of cognitive impairment in late life (Wu et al. 2019). This underscores the importance of addressing the impact of a composite of risk factors rather than focusing on individual factors in isolation. The BCS is a tool that can be easily deployed in primary care and the community to assess individuals’ overall lifestyle habits (Tan and Merchant 2024) and psychosocial risk factors in relation to brain health.

The strengths of this study include a locally adapted score that focuses on the prevention of both dementia and stroke as common age‐related brain diseases. This tool can be used by both patients and healthcare providers in primary and community care. This study adds to the growing evidence of the utility of BCS in promoting brain care and brain health and is the first in an Asian cohort to show the association between BCS and cognitive performance.

There are several limitations to highlight in this study. As a cross‐sectional study, we are only able to examine associations that do not establish causality. Longitudinal studies are needed to examine the longitudinal relationship between changes in BCS and impact on cognitive performance. Second, unlike the validation study in the UK Biobank cohort (Singh et al. 2023), longitudinal data on incident stroke outcome were not available as this was a population‐based questionnaire study. Third, as the BCS is intended as a practical tool for use in primary care rather than an epidemiological examination of its individual components or a predictive model, we did not perform univariable or multivariable analyses on the individual components of the BCS or their associations with outcomes. Thus, we did not account for potential confounders or interactions between the individual components of the BCS. Fourth, impaired recall was defined using a dichotomized cutoff (≤2 words) from the five‐item MoCA recall test. This dichotomization may be influenced by educational, linguistic, or cultural factors and may introduce potential assessment bias. Finally, as a secondary analysis of a population‐based cohort, we were limited by the extent of data collected in the Queenstown baseline study. Nevertheless, we were able to construct a very close approximation of the BCS in this cohort (Table S1). Further studies examining the cultural appropriateness of the BCS and local adaptations are needed.

## 5 Conclusion

In this community‐based cohort from Singapore, higher BCS was associated with better performance in cognitive recall and executive function. These cross‐sectional findings suggest that the BCS may serve as a useful tool for identifying individuals at risk of cognitive impairment in community settings.

## Author Contributions

**Li Feng Tan**: writing – original draft, writing – review and editing. **Jasper R. Senff**: writing – original draft, writing – review and editing, formal analysis. **Shuna Shiann Khoo**: data acquisition. **Sanjula Singh**: writing – review and editing. **Reshma A. Merchant**: conceptualization, writing – review and editing. **Yee Wei Lim**: conceptualization, data acquisition. **Leonard Lee**: conceptualization, data acquisition. **Christopher D. Anderson**: writing – review and editing. **Jonathan Rosand**: writing – review and editing. **Lile Jia**: data acquisition, original draft, writing – review and editing, formal analysis, conceptualization and supervision. **Benjamin Y. Q. Tan**: original draft, writing – review and editing, formal analysis, conceptualization and supervision.

## Funding

The baseline study for Health District @ Queenstown is supported by a grant (E‐581‐00‐0029‐01) from the President's Office, National University of Singapore, awarded to L.J. C.D.A. is supported by RF1NS139183, U01NS069673, American Heart Association–Bugher 21SFRN812095, and the MGH McCance Center for Brain Health. J.R. receives research funding from the US National Institutes of Health and the American Heart Association.

## Ethics Statement

The authors assert that all procedures contributing to this work comply with the ethical standards of the relevant national and institutional committees on human experimentation and with the Helsinki Declaration of 1975, as revised in 2013. All procedures involving human subjects/patients were approved by the Institutional Review Board of the National University of Singapore (NUS‐IRB‐2023‐297).

## Conflicts of Interest

J.R. has consulted for the National Football League and for Eli Lilly, and other authors declare no conflicts of interest.

## Supporting information




**Supplementary Material**: brb371449‐sup‐0001‐SuppMat.docx

## Data Availability

The data have not been previously presented orally or by poster at scientific meetings.
