# Supplementary material for: Association Between Brain Care Score and Cognitive Performance: Findings From a Community‐Based Cohort in Singapore
Source: Brain Behav. 2026 May 8;16(5):e71449. doi: 10.1002/brb3.71449 (PMC13154757; doi:10.1002/brb3.71449)
Supplement: Supplementary file 1 — Supplementary Material: brb371449‐sup‐0001‐SuppMat.docx [file BRB3-16-e71449-s001.docx]

**Table S1: Comparison of Original Brain Care Score (BCS) with adapted Queenstown BCS**

| Category | | McCance Brain Care Score | Rank | Adapted BCS | | rank |
| --- | --- | --- | --- | --- | --- | --- |
| Physical | Blood pressure | Resting blood pressure greater than 140/90, with or without treatment | 0 | “Do you have a high blood pressure/hypertension?” yes | | 0 |
|  |  | Resting blood pressure 120-139/80-89, with or without treatment | 2 | “Do you have a high blood pressure/hypertension?” no | | 3 |
|  |  | Resting blood pressure less than 120/80 | 3 |  |  |  |
|  | Body Mass Index | Obesity ≥30 kg/m2 | 0 | Obesity (≥27.5 kg/m^2^) | | 0 |
|  |  | Overweight 25-29.9 kg/m^2^ or underweight <18.5 kg/m^2^ | 1 | Overweight (23-27.5 kg/m^2^) or underweight (<18.5 kg/m^2^) | | 1 |
|  |  | 18.5-25 kg/m2 | 2 | Normal Weight (18.5-23 kg/m^2^) | | 2 |
|  | Cholesterol | Cholesterol  190mg/dL or higher | 0 | “Do you have a high cholesterol/hyperlipidemia?” yes | | 0 |
|  |  | No treatment required or less than 190 mg/dL  If cardiovascular disease is present, LDL is in accordance to the latest CDC recommendations | 1 | “Do you have a high cholesterol/hyperlipidemia??” no | | 1 |
|  | Diabetes | Hemoglobin A1c greater than 6.4 | 0 | “Do you have Diabetes?” yes | | 0 |
|  |  | Hemoglobin A1c between 5.7 and 6.4 | 1 |  |  |  |
|  |  | Hemoglobin A1c less than 5.7 | 2 | “Do you have Diabetes?” no | | 2 |
| Lifestyle | Nutrition | Dietary habits:  • 4.5 servings of fruit and vegetables per day;  • 2 servings of lean protein per day • 3 or more servings of whole grains per day  • Less than 1,500 mg of sodium per day  • Less than 36 oz of sugar sweet beverages (soda, juice, etc.) per week |  | On a scale from 1 to 5 (never – rarely – sometimes, often – always)   1. Do you eat according to a healthy plate 2. Do you consume things based on healthy eating symbols? 3. Do you consume drinks and beverages with less sugar? | |  |
|  |  | Typical weekly diet does not include at least 2 of the recommendations above | 0 | Average of these 3 questions is less than 3 | | 0 |
|  |  | Typical weekly diet includes ≥2 of the recommendations above | 1 | Average of these 3 questions is 3 to 4 | | 1 |
|  |  | Typical weekly diet includes ≥3 of the recommendations above | 2 | Average of these 3 questions is 4 or more | | 2 |
|  | Alcohol consumption | ≥4 alcoholic drinks per week | 0 | More than once per week | | 0 |
|  |  | 2-3 alcoholic drinks per week | 1 | Once per week | | 1 |
|  |  | 0-1 alcoholic drink per week | 2 | Not drinking or less than once a week | | 2 |
|  | Smoking | Current smoker | 0 | Currently smoking | | 0 |
|  |  | Never smoked or quit more than a year ago | 3 | Currently not smoking | | 3 |
|  | Physical Activity | < 150 minutes of moderate or 75 minutes of high intensity physical activity per week | 0 | I don’t do moderate activities almost everyday or do vigorous activities at least once or twice a week | | 0 |
|  |  | ≥ 150 minutes of moderate physical activity (ex. walking) or 75 minutes of high intensity  physical activity per week | 1 | I do moderate activities almost every day or do vigorous activities at least once or twice a week | | 1 |
|  | Sleep |  |  | On a scale from 1 to 5  #1 (very dissatisfied – dissatisfied, neither, satisfied, very satisfied)  #2 (Very much interfering, much, somewhat, a little, not at all)   1. How satisfied are you with your current sleep pattern 2. To what extent do sleep problems interfere with your daily functioning | |  |
|  |  | Untreated sleep disorder and/or sleeps <7hrs per night | 0 | Average of these 2 questions is 3 or less | | 0 |
|  |  | Treated sleep disturbances and 7-8 hours of routine sleep per night | 1 | Average of these 2 questions is more than 3 | | 1 |
| Social-Emotional | Social relationships | I have few or no close connections other than my spouse or children | 0 | Do you have at least 2 friends or family who you see at leasy once a month, with whom you feel at ease or close? – No | | 0 |
|  |  | I have at least two people, other than my spouse or children, that I feel close with and could talk  about private matters or call upon for help | 1 | Do you have at least 2 friends or family who you see at least once a month, with whom you feel at ease or close? – Yes | | 1 |
|  | Stress | High level of stress that often makes it difficult to function | 0 | I’ve been feeling anxious and nervous often or all of the time | | 0 |
|  |  | Moderate level of stress that occasionally makes it difficult to function | 1 | I’ve been feeling anxious and nervous some of the time | | 1 |
|  |  | Manageable level of stress that rarely makes it difficult to function | 2 | I’ve been feeling anxious and nervous none of the time | | 2 |
|  | Meaning in life |  |  | On a scale from 1 to 5  (strongly disagree, somewhat disagree, neither, somewhat agree, strongly agree)   1. I feel satisfied with the way my life has turned our 2. I have a good sense of what makes my life meaningful 3. Life has been a continuous process of learning, changing and growth | |  |
|  |  | I often struggle to find value or purpose in my life | 0 | Average of these 3 statements is 4 or less | | 0 |
|  |  | I generally feel that my life has meaning and/or purpose | 1 | Average of these 3 statements is more than 4 | | 1 |
|  | | | | | **Total derived BCS ranging** | 0-21 |

**Figure S1: Distribution of BCS across population**


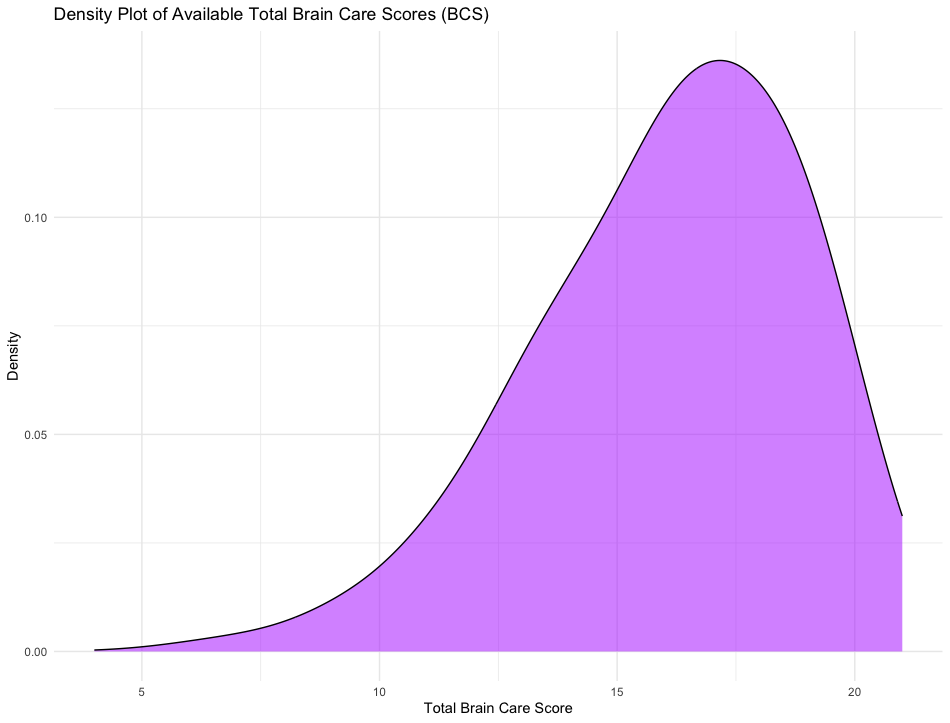


**Figure S2: Distribution of Components across BCS Quartiles**


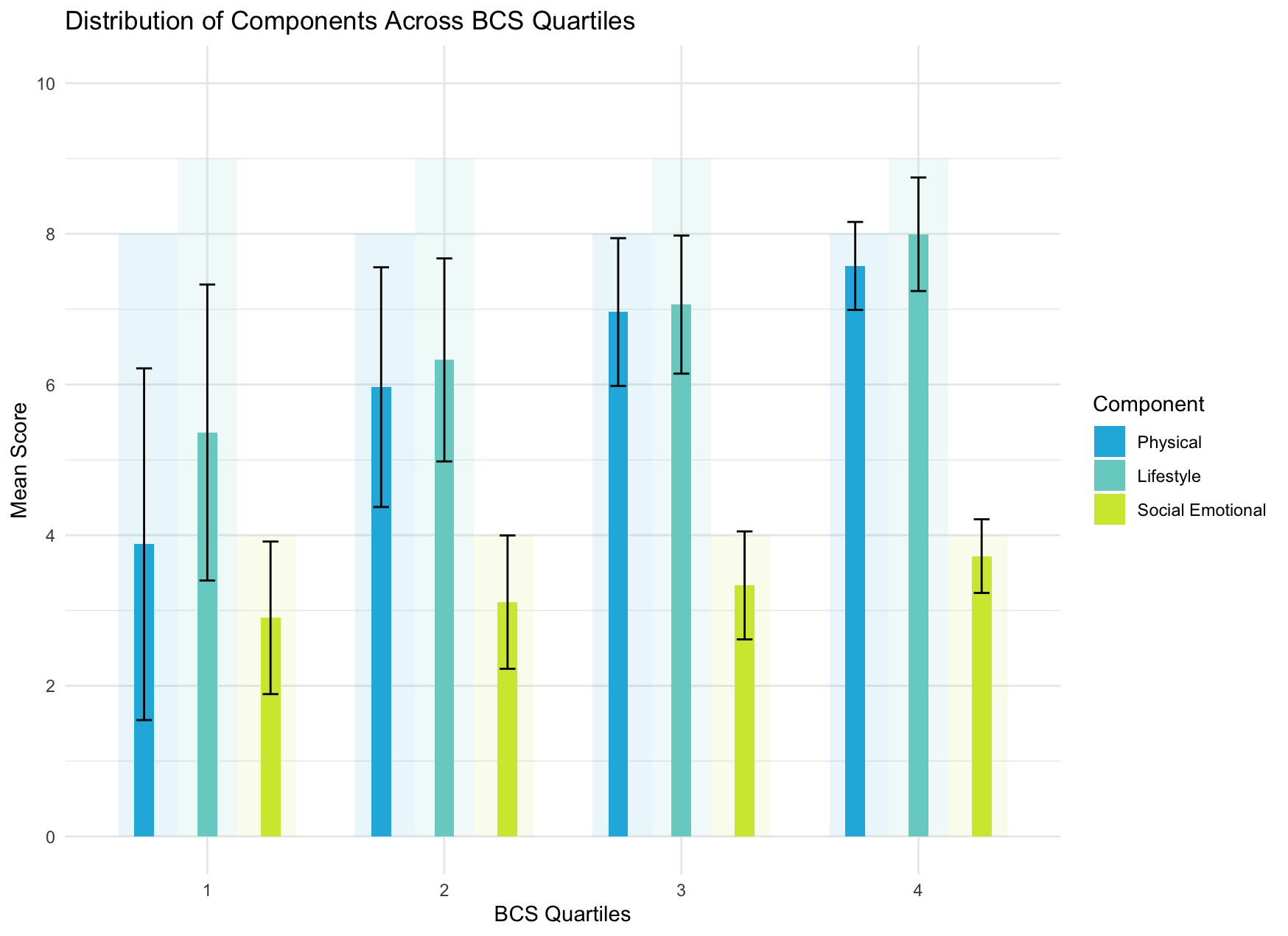


**Figure S3: Gap**

**
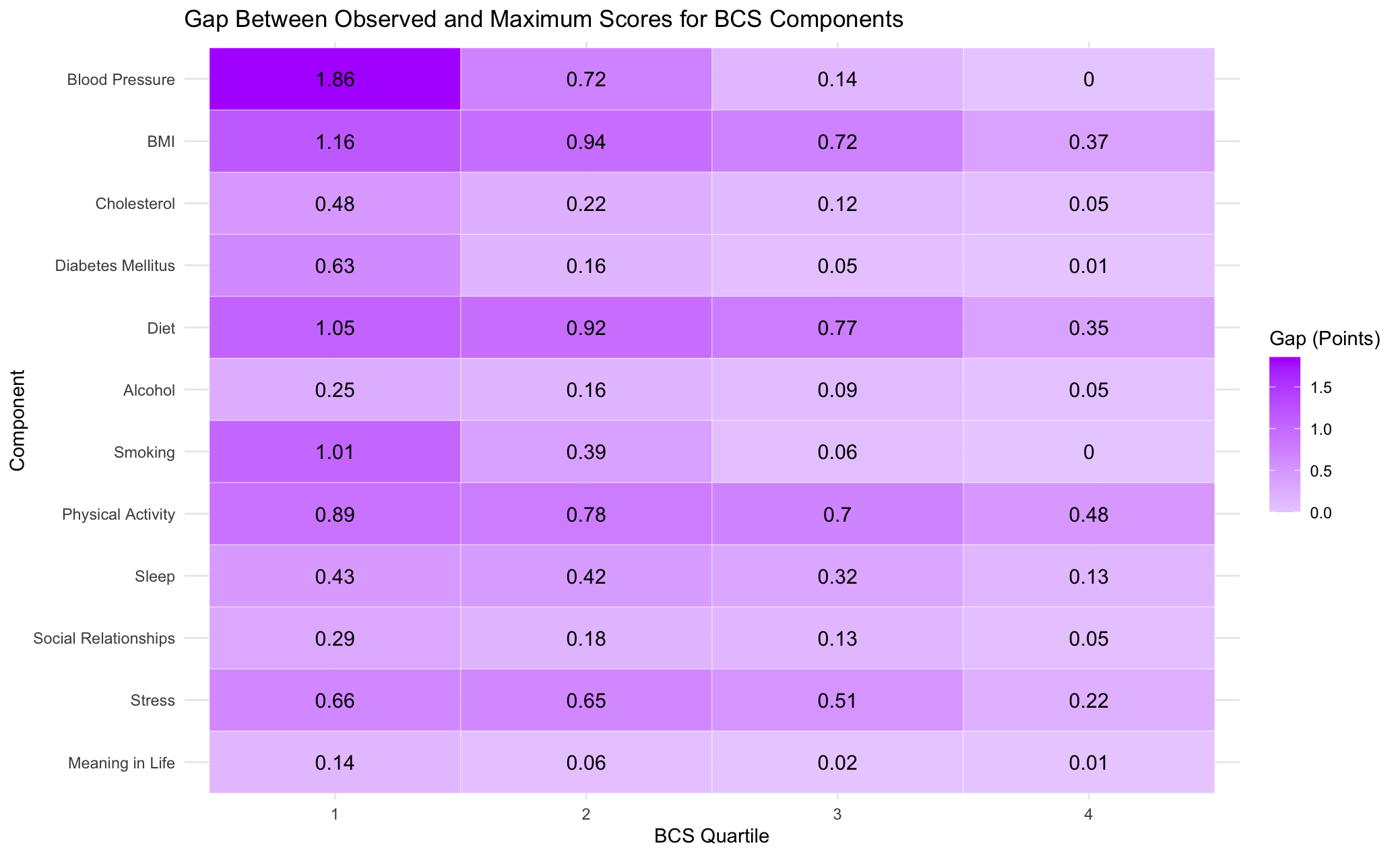
**
